# Locating Insulation Defects in HV Substations Using HFCT Sensors and AI Diagnostic Tools

**DOI:** 10.3390/s24165312

**Published:** 2024-08-16

**Authors:** Javier Ortego, Fernando Garnacho, Fernando Álvarez, Eduardo Arcones, Abderrahim Khamlichi

**Affiliations:** 1 Ampacimon, 28045 Madrid, Spain; 2 Department of Ingeniería Eléctrica, Universidad Politécnica de Madrid, 28012 Madrid, Spain; 3 Laboratorio Central Oficial de Electrotecnia (LCOE), Fundación para el Fomento de la Innovación Industrial (FFII), 28906 Madrid, Spain

**Keywords:** partial discharges, HV substations, insulation diagnosis, partial discharge location, HFCT sensors, standardization

## Abstract

In general, a high voltage (HV) substation can be made up of multiple insulation subsystems: an air insulation subsystem (AIS), gas insulation subsystem (GIS), liquid insulation subsystem (power transformers), and solid insulation subsystem (power cables), all of them with their grounding structures interconnected and linked to the substation earth. Partial discharge (PD) pulses, which are generated in a HV apparatus belonging to a subsystem, travel through the grounding structures of the others. PD analyzers using high-frequency current transformer (HFCT) sensors, which are installed at the connections between the grounding structures, are sensitive to these traveling pulses. In a substation made up of an AIS, several non-critical PD sources can be detected, such as possible corona, air surface, or floating discharges. To perform the correct diagnosis, non-critical PD sources must be separated from critical PD sources related to insulation defects, such as a cavity in a solid dielectric material, mobile particles in SF6, or surface discharges in oil. Powerful diagnostic tools using PD clustering and phase-resolved PD (PRPD) pattern recognition have been developed to check the insulation condition of HV substations. However, a common issue is how to determine the subsystem in which a critical PD source is located when there are several PD sources, and a critical one is near the boundary between two HV subsystems, e.g., a cavity defect located between a cable end and a GIS. The traveling direction of the detected PD is valuable information to determine the subsystem in which the insulation defect is located. However, incorrect diagnostics are usually due to the constraints of PD measuring systems and inadequate PD diagnostic procedures. This paper presents a diagnostic procedure using an appropriate PD analyzer with multiple HFCT sensors to carry out efficient insulation condition diagnoses. This PD procedure has been developed on the basis of laboratory tests, transient signal modeling, and validation tests. The validation tests were carried out in a special test bench developed for the characterization of PD analyzers. To demonstrate the effectiveness of the procedure, a real case is also presented, where satisfactory results are shown.

## 1. Introduction

PD diagnosis using unconventional methods [1] is currently very widely used by utilities to avoid unexpected failures using many different PD techniques and trends [2,3]. The technical specification TS IEC 62478 [4] provides general rules for online PD measurements using electromagnetic and acoustic methods but does not offer any recommendations for insulation diagnosis. The questions to answer when performing a PD diagnosis in a HV substation are the following: How many PD sources are there? What is the insulation defect associated with each one? Where are they located? And what is the criticality of each PD defect?

Recent studies have developed test procedures to analyze the technical performance of PD analyzers for insulation diagnosis using HFCT sensors [5,6,7,8,9]. The procedure referred to in [5] was developed in the European project [10] for the evaluation of different artificial intelligence (AI) diagnosis techniques [11,12,13,14]. To apply this procedure, a special PD calibrator was built [15] that emulates reference PD sources representative of insulation defects in a HV substation with multiple subsystems (AIS, GIS, power transformers, and power cables). This special PD calibrator can generate different PD sources mixed with background noises. The PD signals generated by the PD calibrator are injected at different sites of a test platform designed for qualification purposes [16]. There are four diagnostic characteristics that can be evaluated by means of this characterization procedure [5]: (1) the sensitivity of the PD signal under different noise conditions, (2) automatic PD clustering capability to determine how many different PD sources there are, (3) PRPD pattern recognition of each PD source with an insulation defect, and (4) PD source location along a HV cable system. However, to date, no efficient procedure or evaluation tests have been introduced for determining the HV subsystem (AIS, GIS, power transformer or power cables) in which the PD source is located when it is close to the boundary between two HV subsystems, e.g., when it is at a cable terminal connected to a GIS, which is one of the most difficult and crucial problems to solve during online PD measurements. Typically, misdiagnoses occur when incorrect PD diagnostic procedures are applied. This problem is presented in depth in Section 2. The diagnostic procedure to determine what HV element is involved in the insulation defect is the contribution of this article in comparison to other previous works. The polarity of the PD pulses is the relevant datum to identify the HV subsystem affected by the PD source [17], but the polarity of PD pulses may be lost during the filtering processes of the PD analyzers. In Section 3, the PD diagnostic procedure developed to perform efficient diagnoses is presented. This diagnostic procedure is implemented using an appropriate PD analyzer and multiple HFCT sensors. Section 4 deals with the HFCT sensor requirements for a correct diagnostic procedure and Section 5 is dedicated to the procedure validation. Finally, in Section 6, an onsite case study is presented to corroborate the previous validation and to show the effectiveness of the procedure.

## 2. Analysis of Incorrect Insulation Diagnosis

### 2.1. Common Erroneous Insulation Diagnosis

**Figure 1:**
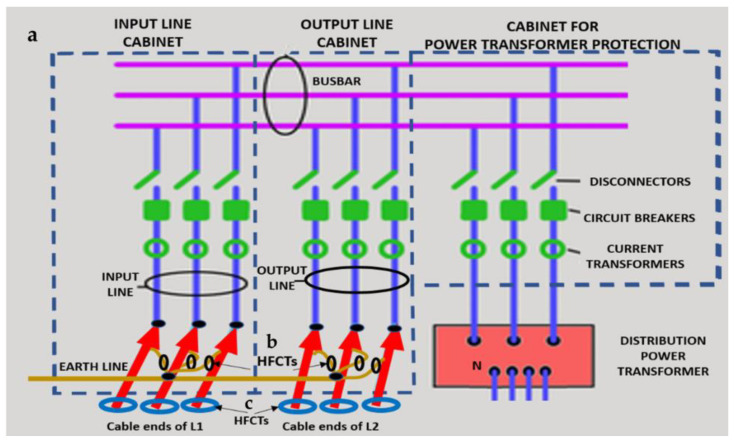
Schematic three-phase circuit of a MV/LV substation: a. general view of the three MV cabinets: MV cabinet of the input line L1, MV cabinet of the output line L2, and MV cabinet of the PTP, b. HFCT sensors hugging the braids of each end of the cables, and c. HFCT sensors hugging each input and output power cable.

A common mistake when performing insulation diagnosis is to correlate the amplitude of the measured PD pulses with the proximity of the fault. The criterion used is that the greater the amplitude, the closer the insulation fault is. This rule is not always true; the pulse amplitude depends not only on the attenuation along the pulse path, but also on the wave impedance values of the different conducting paths, resonances, reflections, etc. The most critical error during online PD measurements is due to the strong dependence of the electrical circuit on the sensitivity of the HFCT sensor, when the disconnectors or switches of the electrical circuit are opened or closed. To demonstrate these errors, a typical MV/LV substation is used, consisting of three MV cabinets interconnected by a common three-phase bus (see Figure 1): one MV cabinet is used for the input line L1, another for the output line L2 (e.g., for the transmission line to the next MV/LV substation) and the third is used for the power transformer protection (PTP). Each MV cabinet is provided with its switchgear: a three-phase disconnector and a three-phase circuit breaker. In most cases, the disconnectors and the circuit breaker are integrated into the same “disconnector-switch” device, hereinafter called “switch”. The current protection transformers give the current signal to the relays to open the circuit breakers in case of overcurrent or an eventual short circuit.

Continuous PD monitoring is usually performed using HFCT sensors installed hugging the grounding braid of each terminal (b in Figure 1), but when the cable ends are not accessible and sporadic PD measurements are required, HFCT sensors hugging the over-sheath of each power cable are used (c in Figure 1).

**Figure 2:**
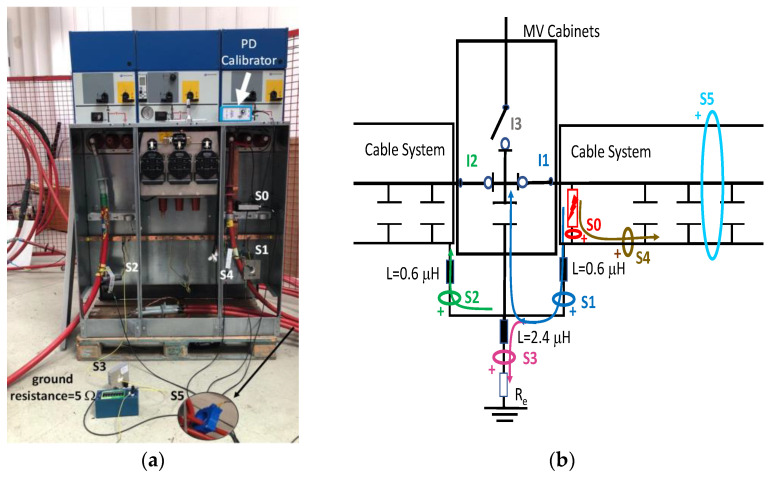
(**a**) Three MV cabins of the emulated MV/LV substation. (**b**) Equivalent circuit of the testing setup.

It would seem reasonable to diagnose a defect inside the MV input cabinet if the HFCT sensors placed on the grounding sheaths of the input cable (see b in Figure 1) HFCT sensors in Figure 1 only measure PD signals after closing the input switch I1, and they do not measure any PD signal when the input switch I1 is open. However, a very similar measurement would be obtained if a cavity defect were in the cable terminal connected to the MV cabinet of the input line L1; this will be justified in Section 2.2. If PD pulses generated by a cavity defect were generated on a input cable terminal L1 connected to the MV cabinet, they would not be detected by any of the HFCT sensors hugging the MV input cables until the input switch I1 was closed. This leads to the common mistake of thinking that the defect is in the MV cabinet, since PD signals are only detected when the input cabinet is energized and are not detected (PD signals apparently disappear) when it is not energized, although PD signals are continuously present in the cable terminal that is always energized. This is justified because the sensitivity of the HFCT sensors is only relevant when a current flows through the stray capacitances of the MV cabinet if the input switch is closed (see sensor S1 in Figure 2b). Additionally, it will be demonstrated in Section 2.2 that HFCT sensors that hug the cable over-sheath (main conductor and cable sheath) always have a lower sensitivity than HFCT sensors placed on the ground braid of the cable terminations.

### 2.2. Laboratory Test of an Insulation Defect in a Cable Terminal Connected to a MV/LV Substation

The laboratory test shown in this section demonstrates a common diagnostic error when performing PD measurements using HFCT sensors that hug the input or output power cables of a MV/LV substation. Three MV cabins of an MV/LV substation were used for the laboratory test, as shown in Figure 2. The input and output MV cabins are provided from switch I1 and switch I2. A single MV power cable was connected to the input cabinet L1, and another was connected to the output cabinet L2 to emulate one phase of a MV distribution network. For simplicity, no MV power cable was used to connect the third MV cabinet to any power transformer (see Figure 1).

For this laboratory test, six HFCT-type sensors were placed in the testing setup (see Figure 2b). Each HFCT sensor was placed with their current arrow pointing in the direction of the earth connection. Sensor S0 was placed at the output of the calibrator to determine the actual injected charge. Sensors S1 and S2 hug the ground braids on both cable ends of the input cabinet L1 and output cabinet L2. Another sensor, S3, was placed in the common grounding connection of the cable sheaths to the laboratory earth of 5 Ω, emulating the real earth resistance of a MV/LV substation. Sensor S4 was placed in the cable sheath at the entrance of the power cable (line L1). The last sensor, S5, was placed hugging the over-sheath of the input power cable L1 (main conductor and its sheath), as shown in the detail circle of Figure 2. When switch S1 is open, sensor S5 measures the differential current between the injected current, S0, and the current pulse returning through the cable sheath L1, S4, which is the most significative part of the injected pulse. Otherwise, when switch I1 is closed, the signal measured by S5 will be even lower than the differential value measured by sensors S0–S4.

The laboratory test was performed by injecting 1000 pC PD pulses with a conventional PD calibrator according to IEC 60270 [18]. PD pulses were injected between the main conductor of the input cable (L1) and its cable sheath. This pulse injection emulates an insulation defect, e.g., in the cable terminal connected to the input cabinet L1. The insulation defect is represented in Figure 2b by a pulsed current source, which is located at the cable terminal of the input line L1. The PD measurement results obtained in three different switching scenarios of switches I1 and I2 are shown in the third column of Table 1: (a) both switches I1 and I2 are closed (MV cabinets and output line cable L2 are energized), (b) switch I1 is open and switch I2 is closed (only input cable L1 is energized), and (c) switch I1 is closed and switch I2 is open (input cable L1 and input cabinet L1 are energized).

(a) Both switches, I1 and I2, closed: The equivalent electrical circuit and the current paths are shown in the second row, second column of Table 1. Sensors S1 and S2 detect a similar signal, but of opposite polarity. The amplitude of both signals is of the order of 45% of the peak value of the injected PD signal S0 (100%). Both sensors S1 and S2 have enough sensitivity for the detection of this defect. Sensor S3 does not detect any significant PD signal. S4 detects a pulse amplitude around 60% of the injected signal because a significant part of the injected PD pulses return through the cable sheaths. The pulse current measured by sensor S5 that hugs the global cable is very low (<4%).
(b) Switch I1 open and switch I2 closed: Sensor S1 only detects an amplitude around 20% of the injected PD pulse (low sensitivity) and S2 and S3 present a negligible sensitivity (<4%) (practically insensitive). This is justified because when the switch is open, it works as a poor capacitor. It would be difficult to detect any defect when the line input switch, I1, opens. Most of the PD pulse current returns through the cable sheath itself, sensor S4 (90%); so, the current measured by sensor S5 is negligible because the injected pulse from the main conductor of L1 cable returns through its own screen.
(c) Switch I1 closed and switch I2 open: Sensor S1 detects an amplitude of the order of 40% of the amplitude of the injected PD pulse S0 (100%) and sensor S2 detects under 5% (practically insensitive). Sensor S1’s sensitivity is justified because a greater capacitive effect appears when the busbars of the MV cabinets are energized. Sensors S3 and S5 present a negligible sensitivity (<4%). Most of the PD pulse returns through the cable sheath being detected by sensor S4 (90%).

### 2.3. PD Source Located near the Boundary between Two Subsystems in a Three-Phase Grid

**Figure 3:**
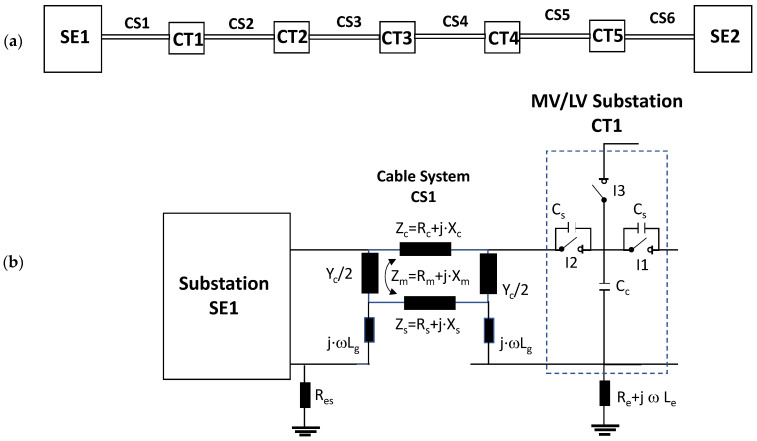
(**a**) Model emulating a set of five MV/LV substations (TC1, TC2,…, TC5) interconnected by 250 m length 12/20 kV cable systems with a 240 mm^2^ aluminium section; (**b**) electrical parameters of each network element used for the transient simulation.

The conclusions of the single-phase test presented in Section 2.2 are extended to a real three-phase energy distribution network through an electrical equivalent model (see Figure 3). This network model emulates a set of five MV/LV substations (TC1, TC2, …, TC5) interconnected by 250 m long 12/20 kV cable systems of a 240 mm^2^ aluminum section (Figure 3a).

For the three-phase transient simulation, the parameters for each element of the equivalent circuit used for the network model (cable systems, MV cabinets with their switchgear, SE substations, and the corresponding grounding systems) are shown in Figure 3. Each cable system is shown in Figure 3 as a quadrupole with five discrete components: a series impedance representing the resistance and inductance of the cable’s main conductor Z_c_ = R_c_ + j X_c_, two parallel admittances emulating the half capacitance Cc/2, the cable sheath impedance Z_s_ = R_s_ + j X_s,_ and a mutual coupling impedance Z_m_ = R_m_ + j X_m_, which are modeled by distributed constants using the Bergeron cable model. These parameters are calculated at a frequency of 250 kHz using ATP (the software used for the transient analysis) from the manufacturer’s data sheet. The parameters calculated by the Carsson formulas used in the ATP software (version 2023) are as follows:

Z_c_ = (2.3326·10^−01^ + j· 2.3006) Ω/m; Z_s_ = (2.3093·10^−01^ + j · 2.1334) Ω/m; Z_m_ = (2.3003·10^−01^+ j · 2.1339) Ω/m

and the admittance value is given by the manufacturer’s data sheet parameters: Y_c_/2 = j4.2252·10^−04^/2 S.

When switches I1 and I2 of each MV cabinet are open, a stray capacitance is considered, whose value was determined by laboratory measurements (C_s_ = 300 pF). However, no stray capacitance was considered for switch I3 because no cable was connected at the cabinet position (protection output) during the laboratory test (see Figure 2a). Furthermore, earthing capacitances between the phase and MV cabinet grounding were modeled by three stray capacitances, which were also determined by laboratory measurements (C_c_ = 550 pF).

The two ends of each cable system were connected to the ground of each MV cabinet through a copper braid of approximately 0.5 m in length, the equivalent inductance of which was estimated at 0.6 μH (1.2 μH/m × 0.5 m). The HFCT sensors S1 and S2 were placed on these copper braids.

Finally, the connection between the cabinet grounding and the MV/LV substation earth point, of around 2 m in length of a flat copper bar strip, was emulated by an equivalent inductance of 2.4 μH (1.2 μH/m × 2 m). The HFCT S3 sensor is placed on this copper bar strip. The earth resistance considered for each MV/LV substation was 5 Ω according to the earth resistance used in the laboratory tests (see Section 2), and the earth resistance in the HV/MV substations SE1 and SE 2 was 1 Ω. The precise final value of each parameter was set to match the simulation waveforms with those of the laboratory test collected in the single-phase test described above in Section 2.2.

A visual comparison between the waveforms shown in the columns corresponding to the “measurements” and “simulation” of Table 1 allows for the observation of a good compatibility between the model and real circuit.

**Figure 4:**
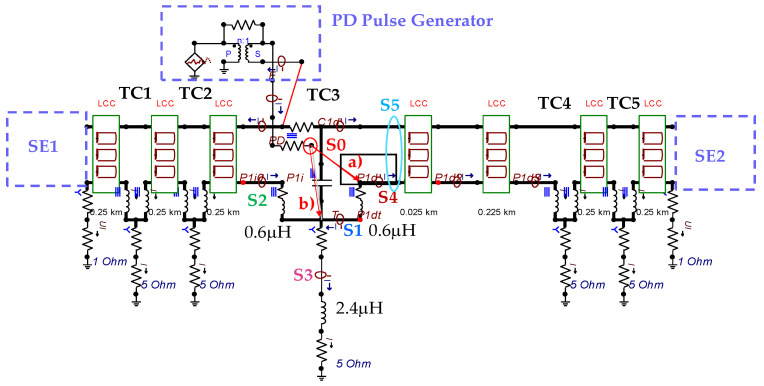
Schematic circuit of ATP for the transient analysis corresponding to the equivalent circuit model shown in Figure 3 when the ideal current pulses are injected: (a) in the cable end and (b) in the MV cabinet in which the cable end is connected.

The insulation defect was generated at the MV/LV intermediate substation “TC3” by means of a simulated PD pulse generator that generates PD pulses 40 ns wide. These pulses are injected at two different sites (see Figure 4) to emulate two different insulation defects: (a) in the cable terminal or (b) into the MV cabinet.

The transient simulation results at the two possible defect locations were obtained for the same three switching scenarios considered for the laboratory test (see Section 2.2), as shown in Table 2. The results provided by the electrical model are compatible with the laboratory test. Additional PD results corresponding to all three phases are reported to analyze the effect of PD pulses traveling in the three-phase circuit. Additionally, a global result for each PD measuring site corresponding to the superposition of the three signals of the HFCT sensors from each site (S1 and S2) has been added, emulating a single global sensor at each measuring site: S1 = S1_U_ + S1_V_ + S1_W_ and S2 = S2_U_ + S2_V_ + S2_W_. Using only one global sensor instead of three sensors (one in each phase) in a PD measurement site has the following two advantages: it is a cheaper solution and provides more sensitive results. The drawback is that it is not possible to determine the phase in which the defect occurs. The S5 signal is considered the subtraction of the pulse current through the main conductor minus the pulse current through the cable sheath, but in practice, due to the magnetic coupling in a ferrite core of an HFCT sensor caused by both currents, it may be different.

The most significant conclusions derived from the results of both emulated defects are the following:

(1) When both switches, I1 and I2 (switching scenario a), are closed, the PD pulse polarity of the signals to be measured by sensors placed at S1 (S1_U_, S1_V_, S1_W_ or S1_U_ + S1_V_ + S1_W_) and at S2 (S2_U_, S2_V_, S2_W_ or S2_U_ + S2_V_ + S2_W_) changes depending on where the defect is. When the defect is in the MV cabinet, the PD pulse polarity of HFCT sensors placed at S1 and S2 is negative, while when the defect is in the cable end of L1 or L2, the polarity of HFCT sensors at S1 or S2 placed in the defective terminal is positive.
(2) The PD sensitivity using only one single global sensor at each measuring site (S1 and S2) hugging the three cable sheaths (S1_U_ + S1_V_ + S1_W_ and S2_U_ + S2_V_ + S2_W_) is better than the sensitivity of HFCT sensors used for each input and output cable sheath (S1_U_, S1_V_, S1_W_ and S2_U_, S2_V_, S2_W_). For example, for switching scenario (a) and the defect in the MV cabinet, the sensitivity of sensor S1_U_ + S1_V_ + S1_W_ is 58%, while the sensitivity of sensor S1_U_ is 48%.
(3) When the input switch I1 is open, the sensitivity of the sensors placed at the S1 and S2 sites is low (<20%). Consequently, no PD conclusions should be stated when switches I1 or I2 are open.
(4) The sensitivity of a sensor placed at S3 is negligible because PD pulses prefer to travel through grounding systems than through the substation earth.
(5) The sensitivity of S5 sensor is not high (<25%). It is not advisable to use HFCT sensors that hug the overall cable (conductor and its cable shield).

## 3. Methodology and Procedure

Identifying the direction of the PD current pulse propagation allows for the discrimination of the affected insulation at the boundary points where a cable connects an MV switchgear, a GIS, or a power transformer. This information is crucial to determine the affected element and to carry out the repair. The PD at their origin presents a Dirac pulse shape with the same polarity as the half-period of the AC voltage applied to the defective insulation.

**Figure 5:**
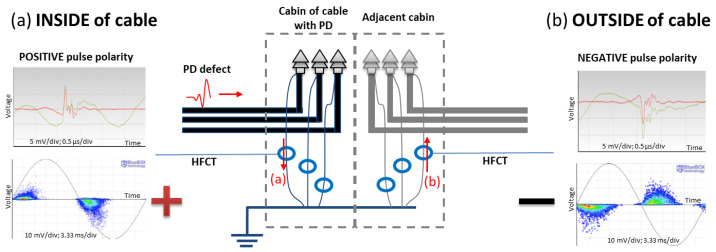
Pulse front polarity of raw signal in green and filtered signal in red, and pattern polarity depending on the traveling direction (**a**) when the PD source is at the cable system side for the HFCT installed at the grounding of the cable terminal and (**b**) when the PD source is outside of the cable at the installation side.

To identify the direction of propagation of the discharges, it is sufficient to place the HFCT type sensors on the terminal earth with their arrow pointing in the direction of the earth and identify the type of edge of each pulse in both half-periods. Once the polarity of the pulses in both half-periods has been identified, it is possible to determine how the PD pulses travel during the positive voltage half-period: whether the discharges travel from the cable conductor to the earth them the current pulses go down (see red down arrow a) linked to the red pulse in Figure 5a) so it is possible conclude the insulation defect is on the side of the cable system from the cable terminal, or otherwise, whether the discharges travel from the earth to the cable conductor, then the insulation defect is located on the other side of the cable terminal (see up red arrow b) linked to the red pulse in Figure 5b).

### 3.1. Flowchart to Determine the Direction of Defect Propagation

**Figure 6:**
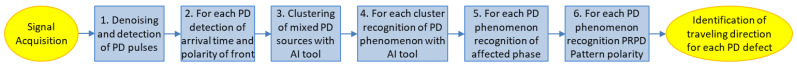
Flowchart of the procedure to determine PRPD pattern polarity.

The procedure for determining the direction of the propagation of the pulses consists in the following steps (Figure 6).

Following this procedure, it is possible to identify each of the PD sources present in the installation and to determine the affected element by means of the following tools described below.

#### 3.1.1. Denoising and Detection of PD Pulses

Denoising is the first challenge of a PD analyzer. Many different denoising tools have been developed for online PD measurements [19,20,21,22,23,24]. The ability to reject non-impulsive noise without losing pulse polarity is analyzed using different filtering methods. The simplest noise rejection method that does not cause the loss of pulse polarity is to reject any signal below the background noise level, but in most cases this method is not able to detect any existing PD signals, as shown in the example of Table 3 (first column). Bandpass filters can remove non-impulsive noise to detect PD pulses, but the efficiency of this approach depends on the chosen measurement frequency range, as shown in the second and third columns of Table 3. For example, for the example case shown in Table 3; selecting the 4 ± 0.5 MHz passband frequency range achieves better filtering than the 8 ± 1 MHz passband frequency range [19]. In both cases, when using a passband filter band, the PD polarity pulse is lost after filtering. A good method to remove non-impulsive noise without losing the original polarity of the pulse is to use a filter based on wavelet transform with statistical analysis [20,21,22]. This filter approach uses the entire bandwidth, while preserving the polarity of the original pulse (see the last column of Table 3).

#### 3.1.2. Detection of Arrival Time and Polarity of a PD Pulse Front

To determine the polarity of the pulse, it is first necessary to identify the time to the peak of the signal and the arrival time. The time to the peak is the time interval between the origin of the pulse and the instant corresponding to the absolute maximum value of the pulse. However, there are several methods to determine the arrival time [25]. In this study, the method based on the energy curve (EC) is used. The energy curve is calculated by means of the following expression:

(1) ECk=∑i=1kyi2−kn·∑i=1nyi2

where

- *n* is the total number of samples that make up the PD pulse;
- *k* is the sample index under consideration to determine the energy curve (EC);
- *i* is the summatory index;
- *y_i_* is the amplitude of the sample *i* in the original PD pulse;
- 1n·∑i=1nyi2 is the average value of the pulse energy.

**Figure 7:**
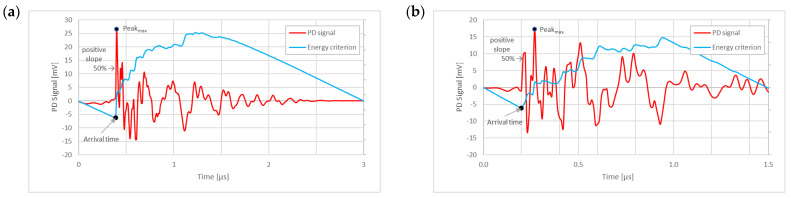
Examples (**a**,**b**) of arrival time identification based on energy criterion and pulse front polarity analysis based on the slope at 50% of the peak.

The energy curve presents a minimum value that is considered the arrival time in which the energy caused by the amplitude of the original PD pulse (first term of Formula (1)) starts to compensate the proportional average value of the pulse energy (second term of Formula (1)), as shown in Figure 7a.

Resonance phenomena can provoke signal superposition, causing peaks up to almost double the initial peak of the pulse.

To avoid confusing the peak of the first front with those of subsequent reflections or oscillations, the identification of the pulse polarity is correlated with the first crossing at 50% of the maximum peak from the arrival time. This method avoids errors with oscillating pulses that have multiple local maxima and minima with very similar absolute values, as shown in Figure 7b.

#### 3.1.3. AI PD Clustering to Separate Mixed PD Sources and Impulsive Noises

(a) PD Clustering by the Analysis of Arrival Time of PD pulses to the Sensors

When performing an online measurement, several PD sources can appear. In these cases, before performing any PRPD pattern recognition, it is necessary to apply automatic PD clustering tools. When using distributed synchronized sensors along the cable system, a first PD clustering is performed by the analysis of the arrival time of PD pulses to the sensors, which permits us to determine the localization as value *x* in meters along the cable section between each pair of synchronized sensors [26,27,28,29]. In this analysis, the arrival time of the PD signals to the sensors is considered, along with the propagation speed of the cable and the distance between sensors. An AI grouping algorithm has been developed to use the localization value *x* for each PD pulse, identifying gaussian distributions over the level of random pairings. For each cluster, the algorithm provides the average localization value of *x* and its standard deviation (*µ*, *σ*). The distance between sensors is inferred from the lengths of the cable sections, and the propagation speed of the cable is estimated by measuring a calibration pulse that is injected into the cable system.

(b) PD Clustering by Identification of the Amplitude Ratio between Phases

Another useful criterion for PD clustering is to determine the phase(s) in which there are higher PD pulses. An extended procedure to determine phase in which there are higher PD pulses is by installing an HFCT sensor at the ground of each phase. The three HFCT sensors should be placed in the same strategic position in the substation, for example, hugging the braid of each cable termination. After applying the filtering tool to the signals acquired by each sensor (see the example shown in the second column of Table 4), the amplitude of each PD pulse “*i*” measured by each phase sensor (*A_Ui_*, *A_Vi_*, *A_Wi_*) is expressed per unit, taking the maximum amplitude of each PD measurement acquisition (*A_imax_*) as the reference value. The three amplitudes in per unit of each PD pulse (*A_ui_*, *A_vi_*, *A_wi_*) are obtained by the following expressions:

(2) $$A_{imax}=\;Max\left(A_{Ui},A_{Vi},A_{Wi}\right)$$

(3) $$A_{ui}=\;ABS\left(\frac{A_{Ui}}{A_{imax}}\right)\;A_{vi}=\;ABS\left(\frac{A_{Vi}}{A_{imax}}\right){\;}_{\;}A_{wi}=\;ABS\left(\frac{A_{Wi}}{A_{imax}}\right)$$

The 3D isometric representation of each PD pulse defined by its three coordinates (*A_ui_*, *A_vi_*, *A_wi_*) is drawn using the U, V, and W axes (see third column of Table 4). Point concentrations on each U, V, and W axis correspond to PD sources located in that phase and point concentrations outside any phase axis correspond to common-mode impulsive noise sources (see last row of Table 4). When only one PD source exists, an AI diagnostic tool [30] can recognize the corresponding PRPD pattern as a specific defect (see example in the last column shown in rows U and V of Table 4), but when more than one PD source is at the same concentration points, as shown in the last column of row W of Table 4, the PRPD pattern cannot be recognized by an appropriate AI tool [19] and an additional clustering approach is required. An additional AI grouping algorithm has been developed to use the isometric representation of this analysis. This is a density-based clustering algorithm using per unit amplitudes (*A_ui_*, *A_vi_*, *A_wi_*) of the ratio analysis presented in the isometric view. For each cluster, the algorithm provides coordinates of the centroid as (*A_ui_*, *A_vi_*, *A_wi_*). The output of the PRPD AI recognition tool, which will be presented in Section 3.1.4, returns the probability of belonging to a list of the possible PD type defects. The highest probability of the AI tool is shown in Table 4 and also in Table 5. The last column of Table 4 shows the PRPD patterns of the PD pulses that have been selected in each phase by the orange square (see penultimate column of Table 4). The PRPD pattern images in the following tables are represented using on the vertical axis the magnitude of the PD, on the horizontal axis the time within the sinusoidal voltage signal, and the colored dots to represent the number of pulses where blue is the minimum and the warmer colors ending in red represent the maximum density.

(c) PD Clustering by Analyzing the PD Pulse Waveform

Additional PD clustering tools need to be applied when multiple PD sources are detected in the same phase. In these cases, the analysis of the frequency components and/or time parameters representative of the waveform of each pulse [31,32,33,34] allows the achievement of efficient PD clustering. The PD clustering tool [33] uses three characteristic pulse parameters of each acquired pulse: the oscillation frequency of the pulse and the two time-constants, *α* and *β*, of the double exponential function enveloping the original PD. The representation of the values acquired by these parameters in a 3D diagram allows the achievement of different clusters of pulses (see Phase W of the example in Table 5). The mathematic model fitted to each pulse corresponds to the following equation:

(4) $$i(t)=\cdot \frac{A}{e^{\alpha \cdot t}+e^{-\beta \cdot t}}\cdot sin\;(\omega \cdot t)\;$$

In this step, another AI grouping algorithm has been developed based on density using the 3D space generated by the parameters of this model. For each cluster, the algorithm provides coordinates of the centroid as (*f*, *α*, *β*).

#### 3.1.4. Phase-Resolved PD Pattern AI Recognition

When only one PD source is present in an online PD measurement, PRPD pattern recognition can be applied [35,36,37,38,39,40] to identify the defect related to this PD source. Depending on the type of insulation defect (internal void, internal surface, floating potential, etc.), a first diagnosis can be made. PD sources related to atmospheric air, such as the corona, floating potential, and surface discharges in the air, are generally less dangerous than PD sources related to other dielectric media, because the insulating medium of air is constantly renewed. However, other PD pulses generated in solid, liquid, and gaseous insulations need to be further analyzed. They can cause a cumulative degradation process. The trend of PD activity versus time, current load, ambient temperature, and humidity provides valuable information to make a maintenance decision (see Table 6) [41]. The developed AI tool is based on a convolutional neural network (CNN). The PRPD pattern in the linear and logarithmic representation has been used as input for the CNN. Both inputs are fed into a feature extraction block following the architecture of a state-of-the-art CNN for image classification. The feature extraction block aims to extract the representative traits that identify the pattern of each class of PD. The output of the data model returns the probability of belonging to each of the possible PD type defects shown in Table 6. In Table 4 and Table 5 in the previous sections, the last column shows the highest probability of the CNN output.

#### 3.1.5. Affected Phase Recognition

The method developed to identify the affected phase is based on the phase distribution of the discharges detected for each defect. The discharges of each of the three phases for the same type of defect are always characteristically distributed 120° apart from the adjacent phases, as shown in Table 7. Once the type of defect has been recognized, it is possible to determine where the characteristic distributions corresponding to each of the three phases should appear by establishing ranges within 360°. For example, a corona effect has discharges distributed around the area of the voltage peak in both half-periods, so that the discharges of phase 1 are shown centered at 90° and 270°, while phase 2 would be centered at 30° and 210°, and finally, phase 3 would be centered at 150° and 330°. The remaining defect types have distributions in different zones, but with the help of a pattern database with thousands of real cases in different installations, their characteristic distribution can be characterized. By determining the mean value of the angular distribution and its characteristic value, it is possible to identify the affected phase.

In order to characterize the distribution, it has been chosen to work with a circular representation of 360°, which allows us to study the distribution continuously at the 0° and 360° extremes (see Table 7). It is common that PD phenomena in their initial states may present activity in only one of the two half-periods or with a strong asymmetry in the total of discharges between the two half-periods. To eliminate this casuistry, a change in the variable has been made in the characterization method to simplify the representation by superimposing the two half-periods. This change in variable is achieved by replacing the angle φ at which the discharge occurs by the angle φ’ according to Equation (5). Once the change of variable has been made, we can characterize the distribution at φ’ by calculating the circular mean $\bar{\varphi ’}$ with Equation (6).

(5) $$\varphi ’=\left\{\begin{array}{r} 2\cdot \varphi ,\;\;\;\;\varphi \leq 180{}^{\circ}\\ 2\cdot (\varphi -180{}^{\circ}),\;\;\;\;\varphi >180{}^{\circ} \end{array}\right.$$

(6) $$\bar{\varphi ’}=atan2\left(\sum_{i=1}^{n}\sin{\varphi ’}_{i},\sum_{i=1}^{n}\cos{\varphi ’}_{i}\right)$$

where

- *n* is the number of discharges of the defect;
- function atan2 is defined by next Equation (7)

(7) $$atan2\left(x,y\right)=\left\{\begin{array}{c} \begin{array}{c} \arctan\left(\frac{x}{y}\right),\;\;\;\;\;\;\;\;\;\;\;x>0\\ \arctan\left(\frac{y}{x}\right)+\pi ,\;\;\;\;\;\;\;\;\;\;\;x<0\;\mathrm{and}\;y\geq 0\\ \arctan\left(\frac{y}{x}\right)-\pi ,\;\;\;\;\;\;\;\;\;\;\;\;\;x<0\;\mathrm{and}\;y<0\\ +\frac{\pi }{2},\;\;\;\;\;\;\;\;\;\;\;\;\;\;\;\;\;\;\;\;\;\;\;\;\;\;\;\;x=0\;\mathrm{and}\;y>0\\ -\frac{\pi }{2},\;\;\;\;\;\;\;\;\;\;\;\;\;\;\;\;\;\;\;\;\;\;\;\;\;\;\;\;\;x=0\;\mathrm{and}\;y<0\\ \mathrm{undefined},\;\;\;\;\;\;\;\;\;\;\;\;\;\;\;\;\;\;\;\;\;\;\;\;\;\;\;\;\;\;\;\;\;\;x=0\;\mathrm{and}\;y=0 \end{array} \end{array}\right.$$

The affected phase is determined by calculating $\bar{\varphi ’}$ of the pattern and analyzing which of the three sectors it is located in, corresponding to the three phases. These sectors each cover 120° and have been previously calculated from a database of thousands of defects previously classified manually by diagnostic experts (see Table 7), that are called the three phase 120° sectors.

#### 3.1.6. PRPD Polarity Identification

In the final step, the polarity of the set of pulses forming the defect pattern must be calculated. A pulse by itself could give rise to errors when determining its polarity, due to background noise that is not correctly eliminated, resonance phenomena in the measurement chain, or the overlapping of system reflections. These errors are more significant with lower amplitude pulses, as can be seen in the image of the pattern in Table 8 and the table in Section 6.1, where the PD pulses closest to the base appear in both polarities in both half-periods. To improve the reliability of polarity determination, only the subset of PD pulses whose amplitude value is between the 50th and 100th percentile is used. The method implemented consists in counting the number of pulses that coincide with the polarity of the half-period in which they are found and calculating the ratio of *R_positives_* to the total number of pulses according to the following equation.

(8) $$R_{positives}=\sum_{i=1}^{n}\left\{\begin{array}{c} 1,\;\;a_{PPS}\leq \varphi \leq a_{PPS}+180{}^{\circ}\\ 0,\;\;rest\;of\;cases \end{array}\right./n$$

where *n* is the number of pulses above the 50th percentile of the amplitude of the pattern and $a_{PPS}$ is the starting angle of the area where the positive pulses of the positive half-period of the voltage start, which has been previously characterised using the database mentioned in the previous step.

The resulting *R_positives_* ratio shall determine the polarity of the defect according to the following criteria.

## 4. HFCT Sensors

Currently, HFCT sensors are sensors commonly used for online PD measurements, but their technical characteristics are not well known.

The transfer impedance of a HFCT sensor, defined as the output voltage of the sensor divided by the input current when the output is loaded with a 50 Ω impedance (expressed in mV/mA), should be flat in measuring the frequency range of interest (from 1 MHz to 30 MHz). Otherwise, the PD signal will be distorted and affect the linearity, depending on the frequency content of the signal. The transfer impedance of commercial HFCT sensors is usually in the range between 4 Ω to 15 Ω. Sensors with a high transfer impedance (>8 mV/mA) do not usually have a flat response in the frequency band, so they significantly distort the wave shape of the measured PD pulses. In contrast, sensors that constantly maintain their transfer impedance in the measuring frequency range present a low gain (<5 mV/mA). The higher number of spires wounded on the ferrite toroid core to achieve a high gain requires a winding with a lower inductive component and an appropriate selection of the ferrite material [42].

Another general issue concerning the HFCT sensors used for the continuous monitoring of HV installations is that they have a single winding wounded on the ferrite toroid to measure PD pulses. However, due to stress in HV networks, such as short circuits, surges due to switching, or lightning over-voltages, the ferrite core of these HFCT sensors can suffer premature degradation and consequently lose their gain. This can be unnoticed by the user of the sensor, which would be ineffective for any PD detection. A complementary winding wounded on the same ferrite coil allows for checking the correct operation of the HFCT sensor.

Furthermore, HFCT sensors that are used to monitor the PD in HV installations are under high electric fields, in the order of tens of volts per meter. Therefore, it is very convenient to design a metal shielding to guarantee the correct electromagnetic immunity. However, many of the HFCT sensors on the market do not have any shielding, which directly affects the interference voltage induced in the measuring winding, up to an order of magnitude higher than that of the acquired PD signal. The existing shielded HFCT sensors use a copper or aluminum casing painted or coated with an insulating layer. These shields may be effective in terms of electromagnetic immunity, but they are deficient from an electrical insulation point of view, since an abrasion or degradation of this insulating coating layer can lead to a dangerous voltage in its shielding and consequently on the wire of the measuring winding, endangering the integrity of the measuring instrument and even the safety of the operator who handles it.

Therefore, HFCT sensors must be capable of achieving a flat transfer impedance (e.g., not less than 8 mV/mA) in the frequency measuring range (from 0.5 MHz to 20 MHz), a second winding to check its correct operation, an appropriate shielding for immunity to electromagnetic interferences, and suitable electrical isolation for safety reasons, all in a low-cost format.

With a required transfer impedance of 8 Ω and assuming a peak voltage sensitivity of the PD analyzer digital recorder of at least 0.4 mV, the lowest magnitude of the apparent charge, *q_min_*, to be detected would be in the range between 1.9 pC and 19 pC, depending on the PD pulse width (*T_PD_* = 37.5 ns or 375 ns):

$$q_{min}(for\;shorter\;pulses\;\;T_{PD}=37.5\;\mathrm{ns})=U_{min}\cdot \frac{T_{PD}}{Z_{s}}=0.4\;mV\cdot \frac{37.5\;ns}{8\;mV/mA}=1.9\;pC$$

$$q_{min}(for\;longer\;pulses\;\;T_{PD}=375\;\mathrm{ns})=U_{min}\cdot \frac{T_{PD}}{Z_{s}}=0.4\;mV\cdot \frac{375\;ns}{8mV/mA}=19\;pC$$

For continuous PD monitoring, a PD sensitivity of 20 pC is a reasonable magnitude.

## 5. Validation Test of the Capability to Identify the Defective Element

### 5.1. Testing Procedure

**Figure 8:**
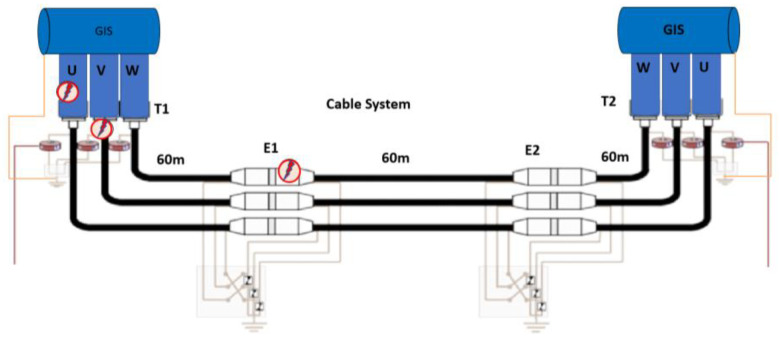
Schematic circuit of the scale model representing a GIS and cable system to check the PD location capability of any PD analyzer using HFCT sensors.

This testing procedure for the characterization of PD analyzers does not require the application of HV, but instead uses a low voltage test setup. This test setup consists of two basic infrastructures: a synthetic PD calibrator [15] capable of reproducing PD pulse trains like those generated in real HVAC installations and a three-phase scale model [16]. The scale model consists of a three-phase coaxial cable system connected to two GIS scale modules, one at each cable end. The cable is 180 m in length and simulates a three-phase cable system in a cross-bonding configuration with three sections of 60 m (see Figure 8). The scale model is designed to place HFCT type sensors in each cable accessory (cable ends and splices). In addition, in each cable accessory, there are three BNC-type connectors to inject PD pulse signals with the synthetic PD calibrator, emulating representative insulation defects with superimposed noise signals. The PD injection can be performed at 18 possible injection points in the cable system (1 per each terminal × 6 terminations + 2 per each splice × 6 splices = 18), and in each GIS in the three phases (3 per each GIS × 2 GIS = 6 injection points). In total, 24 injection points are available. The attenuation of the coaxial cable in the 1 to 10 MHz range is in the order of 10 times greater than that of the expected attenuation of a HV power cable, so the amplitude of the PD pulses will be significantly 10 times reduced when traveling 60 m in each cable section, thus simulating an equivalent length of 600 m. The propagation speed of the coaxial cable is around 15% higher than that estimated for a HV power cable, which is of the order of 169 m/μs. Both parameters, together with the distance between sensors, must be considered during the validation tests related to the PD location along the cable system, as was stated in Section 3.1.3.

The target of the tests to be carried out is to analyze the effectiveness of a PD analyzer using HFCT sensors when there are three PD sources in different locations of the test setup. One is injected in phase U of the GIS, another in phase V of the cable terminal connected to this GIS, and the third in the junction E1 of phase W, located at 60 m from the GIS (see Figure 8). All the defects are generated with the synthetic PD pulse calibrator using a digital database of PD sources representative of insulation defects in GIS, AIS, and cable systems. PD trains are injected to ensure that the HFCT sensors receive detectable signals from the PD sources. The defects coming from the GIS can be associated with a GIS or AIS type. In this test case, PD pulses propagated from the AIS part or generated in the GIS part are simulated by type 1 to type 8 defects shown in Table 6. The defects coming from the cable only correspond to cable defects (types #15 and #16). The cable system is supposed to be long enough so that any signal coming from one substation will be completely attenuated at the other cable end. The test was repeated twice with different PD sources.

### 5.2. Test Results

In this test, the PD analyzer under validation applies a PD clustering approach based on the isometric 3D PDR tool to identify the affected phase/s by defects. After determining the defects located in each phase, the pulse polarity clustering tool is applied to determine whether the sources are in the cable system or in the GIS. The test results are shown in Table 9. This test was repeated twice. In both cases, the three insulation defects were satisfactorily located. Furthermore, they were identified using the AI tool for PRPD pattern recognition. With the pulse polarity tool and the PRPD pattern recognition tool of the PD analyzer under validation, the determination of whether the defect was in the GIS or in the cable termination was possible.

## 6. Case Study of Locating a Real PD Defect in a 12 kV Distribution Grid

**Figure 9:**
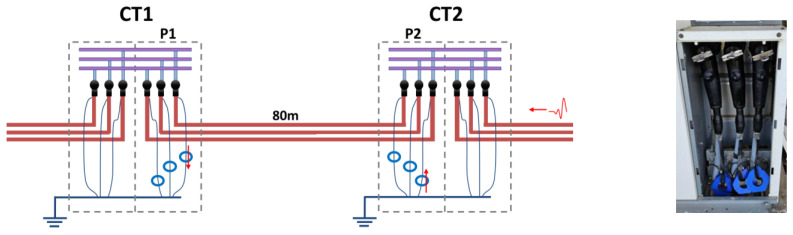
On the left, schematic circuit of the MV cable monitored; on the right, detailed view of HFCT sensors installed hugging the braids of cable terminations. The pulse polarity described in Section 6.2 leads to the red up and down arrow symbol corresponding to the PD pulse currents.

An insulation defect was detected during an online PD test in a 12 kV cable system connecting several MV/LV substations (see Figure 9). After the detection of an insulation defect, locating and repairing the affected element before failure is not a trivial task. In this real experiment, the new procedure demonstrates how the position of the defect at a boundary point can be determined and located by planning further measurements based on the results of the polarity analysis.

The initial measurement (#1) was carried out using synchronized HFCT sensors installed at the terminations of two consecutive MV/LV substations, CT1 and CT2 (see Figure 9). These substations were connected by an 80 m long MV cable system through the cabinet P1 of substation CT1, and through the cabinet P2 of substation CT2. In the acquisition process, the PD analyzer used is responsible for collecting the simultaneously acquired raw signals and for filtering the background noise, without losing the polarity of the pulses. Then, the pulse parameters, including the arrival time, amplitude, phase angle, and those related to the waveform (*f*, *α*, and *β*) of Formula (4), are determined.

### 6.1. Results of AI Clustering and AI Pattern Recognition Tools

During the measurements, several PD phenomena were present in the MV grid, generating a raw PRPD pattern that is difficult diagnose without applying any PD clustering in advance (see the first column in Table 10). In this measurement, multiple defects in different phases were detected. To simplify the demonstration, only one of the internal defects is analyzed step by step, showing the procedure of using the AI tools.

A first set of PD clusters named LOC_P2_C (see in Table 8 the second column) was generated by two AI clustering tools: one based on localization using the arrival time of PD pulses to the HFCT sensors ((a) in Section 3.1.3) and the other based on the amplitude ratio between the three phases ((b) in Section 3.1.3). This set of clusters contains all the defects coming from the cable terminal of MV cabinet P2 or from the rest of the MV grid. A high sensitivity was detected by the HFCT sensor placed at phase C (brown sinusoidal wave). To analyze the pulse polarity and determine if the PD is related to the cable system or to the MV cabinet P2 under testing, an additional PD clustering is needed to separate multiple phenomena that could be mixed in the PRPD pattern. The final cluster, named VOID_P2_C, is obtained with an AI clustering tool based on the analysis of the pulse’s waveform parameters (*f*, *α*, and *β*), according to Formula (4). The AI recognition tool determined that this cluster is representing an internal void PD phenomenon, which will be the subject of in-depth analysis.

The defect type with the highest probability is obtained at the output of the recognition neural network. Table 10 shows the clustering results using the AI tools and indicate the reference values for each cluster in brackets, standing for the location, by means of the mean distance of the PD source *μ* = 78 m, and its standard deviation *σ* = 11 m; for the ratio between the three phases and the isometric coordinates (*A_ui_*, *A_vi_*, *A_wi_*) of the cluster centroid; and for the three waveform parameters (*α*, *β*, and *f*), the coordinates of the cluster centroid.

### 6.2. Recognition of the Affected Phase and Pattern Polarity

**Figure 10:**
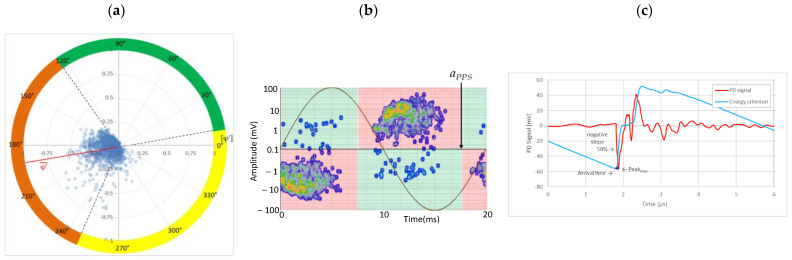
(**a**) Affected phase analysis, (**b**) pattern polarity analysis, and (**c**) pulse polarity analysis in the positive half-period.

Applying the developed method to recognize the affected phase on the set of pulses clustered in VOID_P2_C, it was determined that the affected phase is C (third phase). In this case, the affected phase coincides with the phase of the sensor detecting the highest signal ratio. However, it is not always like that, due to the poor sensitivity of a certain sensor caused by measurement chain failures or by groundings of MV terminations with a bad connection. Figure 10a shows the analysis of the φ’ distribution with a circular mean value $\bar{\varphi ’}=1$89.9°, corresponding to the phase C section for internal void distributions. The polarity of the pattern based on the pulse polarity previously obtained (see Figure 10c) is calculated with the new developed method (see Section 3.1.5). The regions for positive pulses in the PRPD pattern are defined by the angle *a_pps_* characteristic for internal void distributions (see Figure 10b). The ratio of positive pulses is *R_positives_* = 9.3% < 40%, which means that the pattern polarity is negative in this case. This means that the insulation defect is not in the MV cabinet P2 of the MV substation CT2 or in the rest of the grid behind it according to explanation given in Section 3 (see Figure 5).

### 6.3. Measurement #2 Conclusions and Additional Steps for Localization

After applying the complete procedure to the first measurement using HFCT sensors located in P1 and P2 cabinets, the conclusion is that void defect exists related with phase C and comes from the MV cabinet P2 or from behind the rest of the installation.

**Figure 11:**
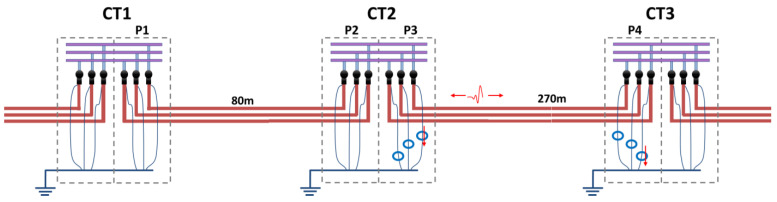
Schematic circuit of the adjacent MV cable system supervised in measurement #2.

An additional measurement was required, and was performed between the MV substations CT2 and CT3, using the AI tools. Pulse polarity in this measurement was carried out in the adjacent 270 m cable system that connects both substations through the MV cabinets P3 and P4, as shown in Figure 11.

Repeating the complete procedure with the online monitoring performed between the MV cabinets P3 and P4 of the MV substations CT2 and CT3, respectively, the internal void defect was detected and located near the MV cabinet P3. Table 11 shows the clustering of the PD detected by the sensor positioned in phase C at the MV cabinet P3. In this case, it was not necessary to apply the PD clustering of waveform parameters because there were no additional defects located at the cable terminal of the MV cabinet P3. The current pulses in both HFCT sensors of phase C located in cabinets P3 and P4 are positive (down arrow), therefore the insulation defect is located in this cable section between CT2 and CT3.

Table 11 shows the clustering results using the AI tools for this second PD measurement, standing for the location by means of the mean distance of the PD source *μ* = −1 m with its standard deviation *σ* = 12 and the ratio between the three phase amplitudes in per unit (*A_ui_*, *A_vi_*, *A_wi_*). Applying the affected phase recognition (Section 3.1.5), the result for this insulation defect is located in phase C, with a positive pattern polarity positive for the sensors installed in both cable ends of the MV cabinets P3 and P4, because PD pulses were traveling from the MV cabinet P3. The In conclusion, the defect was positioned in the cable terminal of phase C connected to MV cabinet P3.

## 7. Conclusions

The insulation condition diagnosis when online PD measurements are performed in HV substations that include several insulation subsystems (air insulation, gas insulation in switchgear, solid insulation in power cables, and oil insulation in power transformers) entails a great deal of difficulty. Incorrect diagnoses are likely to be made when a defect is near or at the junction point between a cable terminal and a metal-enclosed switchgear. Common diagnostic errors have been analyzed through laboratory testing and simulations using transient signal modeling. It has been shown that most errors occur when the sensitivity of HFCT sensors decreases, depending on the switch position in the HV substation (open or closed). For example, the sensitivity of HFCT sensors that hug the braids of cable terminations connected to a metal-enclosed switchgear is only good enough when the switchgear is energized. If it is not energized, the sensitivity is very low, even for an HFCT sensor positioned at the cable terminal where an eventual PD defect could be located. However, when the metal-enclosed switchgear is energized, PD pulses can be better detected, including those coming from the cable system and those coming from the metal-enclosed switchgear. The polarity analysis of PD pulses versus the applied voltage has been shown to be an effective way to determine the correct location of the insulation defects.

A new method has been introduced for insulation diagnosis using HFCT sensors, with which the pulse polarity of the same group of the PD is identified. In the first step of this diagnostic procedure, a filtering tool, based on the wavelet transform, is used to remove non-impulsive background noise signals present in the HV installation. After applying this noise filtering tool, the polarity of the original pulses remains unchanged. Bandpass filters can change the polarity of the original PD pulses, while wavelet transform filters preserve it. In the second step, a robust procedure to avoid false polarity identification due to signal overlap caused by signal reflections is applied. Reflection phenomena can cause secondary oscillations in the PD signals, with amplitudes larger than those of the first oscillation. If the polarity is correctly determined, the element affected by the defect can be easily identified. In the third step of the procedure, different clustering approaches are used to identify the affected phase, the number of PD sources, and their location. In the last step, the insulation defects are identified by analyzing the PRPD patterns using an efficient artificial intelligence tool. The diagnostic method shown in this paper has been validated by performing various tests on a scale model that simulates a GIS and a power cable system, and using a synthetic calibrator that reproduces PD sources representative of real defects. The positive validation results obtained with the scale model and the synthetic calibrator were ratified with an onsite case study, thus demonstrating the effectiveness of the method proposed in this research.

## Figures and Tables

**Table 1:** PD pulses measured in the laboratory test when there is a localized insulation defect at the end of the cable (emulated by injecting 1000 pC PD pulses generated by a PD pulse calibrator).

Switching Scenarios	Defect in	Signals Referred to Injected Signal (Reference Signal S0 = 100%)	
		S0, S1; S2; S3; S4; S5	
		Measurements	Simulation
(a) I1 and I2 close	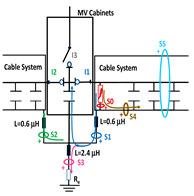	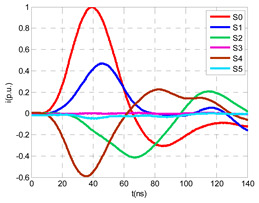	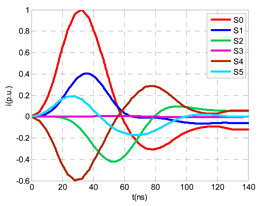
(b) I1 open and I2 close	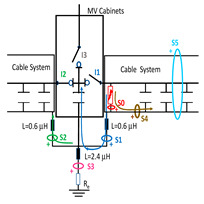	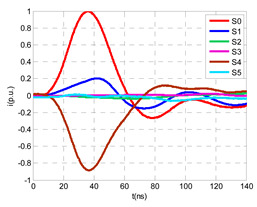	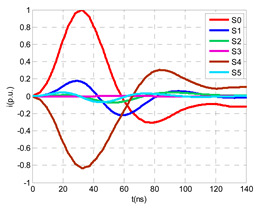
(c) I1 close and I2 open	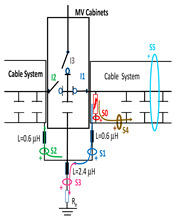	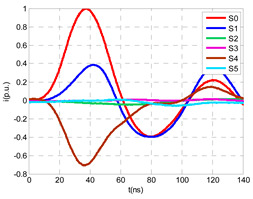	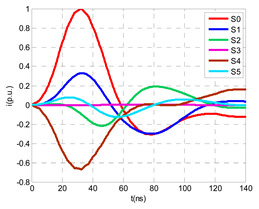

**Table 2:** Simulation results of PD pulses at the sensor sites when two insulation defects are emulated by means of the injection of PD pulses of 1000 pC.

Switching Scenario	Defect in	S1_U_, S1_V_, S1_W_ S2_U_, S2_V_, S2_W_	S1_U_ + S1_V_ +S1_W_ S2_U + V+W_	S3 S4_U_, S4_V_, S4_W _ S5_U_, S5_V_, S5_W_
(a) I1 and I2 close	Cable termination 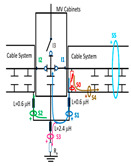	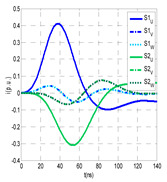	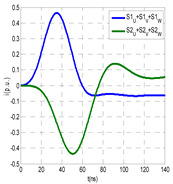	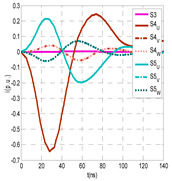
	MV Cabinet 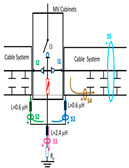	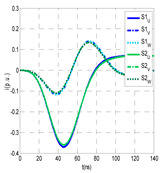	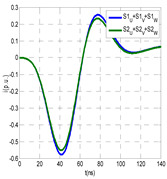	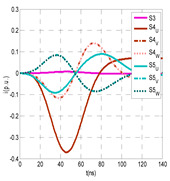
(b) I1 open and I2 close	Cable terminal 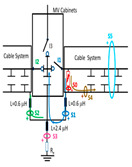	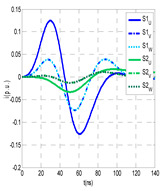	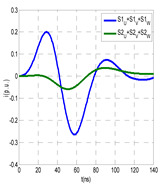	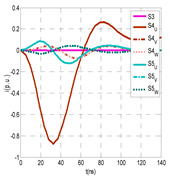
	MV Cabinet	No PD activity		
(c) I1 close and I2 open	Cable termination 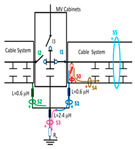	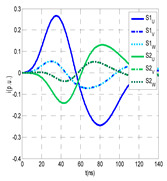	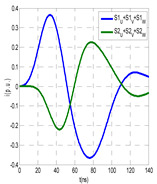	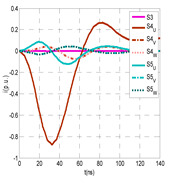
	MV Cabinet 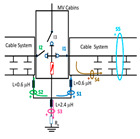	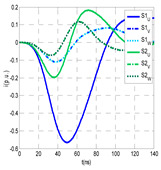	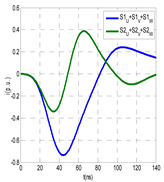	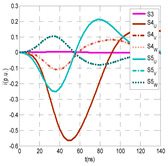

**Table 3:** Different filtering approaches to remove non-impulsive noises: (a) selecting the threshold level, (b) 8 ± 1 MHz bandpass filter, (c) 4 MHz ± 0.5 MHz bandpass filter, (d) wavelet transform and statistical analysis.

Full Bandwidth + Selection of the Threshold Triggering Level	Band Pass Filter (8 MHz ± 1 MHz) + Threshold Level	Band Pass Filter (4 MHz ± 0.5 MHz) + Threshold Level	Full Bandwidth + Wavelet + Automatic Statistical Analysis
** 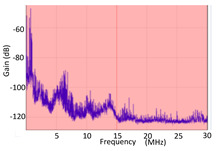 **	** 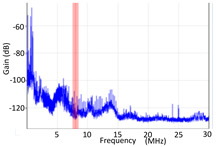 **	** 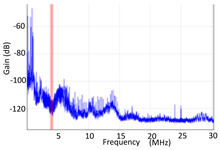 **	** 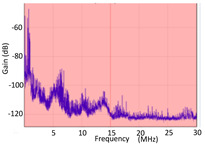 **
PRPD Pattern after threshold selection	PRPD Pattern after Band pass filter (8 ± 1 MHz)	PRPD Pattern after Band pass filter (4 ± 0.5 MHz)	PRPD Pattern after filtering Wavelet + statistical analysis
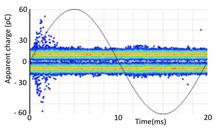	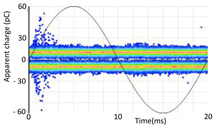	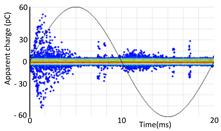	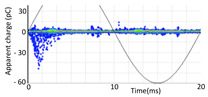
Manual PRPD Pattern	PRPD pattern after noise threshold removal	PRPD pattern after noise threshold removal	Automatic PRPD Pattern
No detection	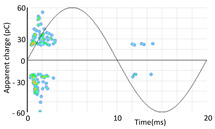	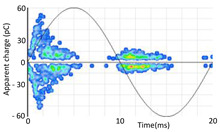	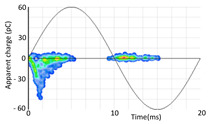

**Table 4:** PD clustering by means of isometric 3D representation of each PD pulse (*A_Ui_*, *A_Vi_*, *A_Wi_*).

Sensor Phase	PRPD Measured by Each Phase Sensor at a Substation Position	Isometric 3D PDR (*A_Ui_*, *A_Vi_*, *A_Wi_*)	Clustering by Phase Selection (Orange Square)	PRPD of Pulses Related to Each Phase (Only PD Pulses in Orange Square) + AI Tool for PRPD Pattern Recognition
U	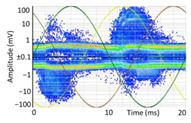	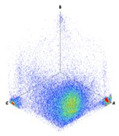	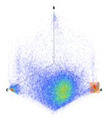	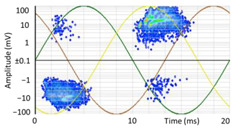 Type defect recognized: internal void (93%)
V	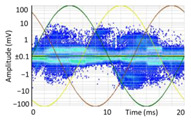		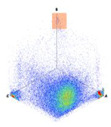	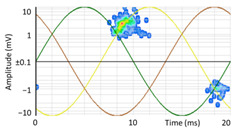 Type defect recognized: Internal Surface (95%)
W	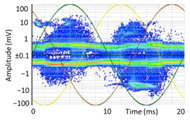		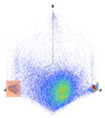	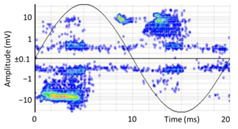 No kind defect is recognized by the AI tool
Impulsive noises in common mode			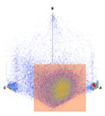	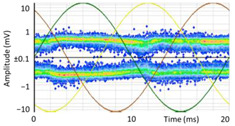

**Table 5:** Additional PD clustering by analyzing the PD pulse waveform applied to the pulses related to phase W.

PD Clusters Related to the Phase W	PRPD Pattern of the Pulses Related to the Phase W	AI Tool for PRPD Pattern Recognition
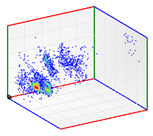	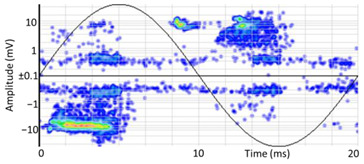	No PRPD pattern recognition
Selection of the cubic 3D space defined in orange color	PRPD pattern of the selected orange cubic 3D space	Recognition of the defect type applying an AI tool
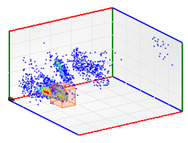	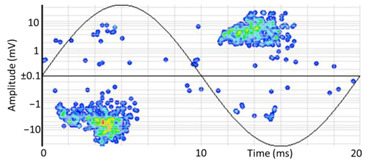	Internal void (93%)
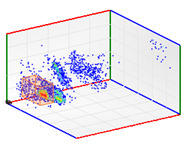	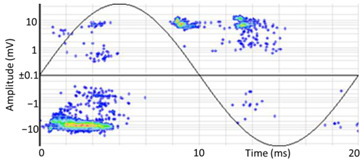	Floating potential (96%)
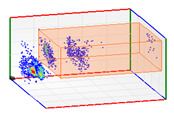	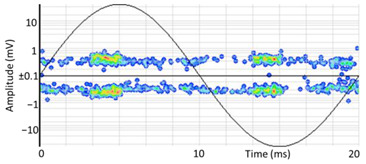	Impulsive noise (99%)

**Table 6:** Examples of PRPD patterns associated with different insulation defects whose criticality depends on the affected subsystem with a specific insulation medium affected.

Subsystem	AIS	GIS	Power Transformer	Cable System
Corona or Protrusion	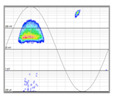 in air (type#1)	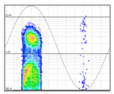 in SF6 (type#4)	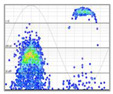 in oil (type#9)	It is not possible
Surface	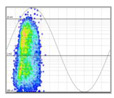 in air (type#2)	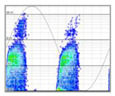 in SF6 (type#5)	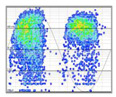 in oil (type#10)	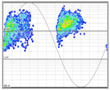 Surface between solid insulations (type#15)
Mobile particles	It is not possible	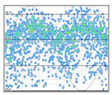 in SF6 (type#6)	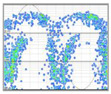 in oil (type#11)	It is not possible
Floating Potential	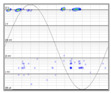 in air (type#3)	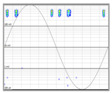 in SF6 (type#7)	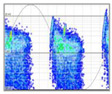 in oil (type#12)	It is not possible
Void	It is not possible	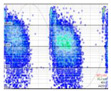 in a spacer (type#8)	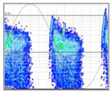 in paper oil (type#13)	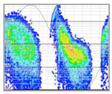 in XLPE (type#16)
Burbles	It is not possible	It is not possible	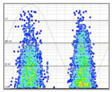 in oil (type#14)	It is not possible

**Table 7:** Characterization of pulse distribution by phase.

Phenomenon	PRPD Pattern	Circular Distribution of *φ*	Circular Distribution in the three phase 120° sectors with Change of Variable *φ’*
Corona Effect in phase 1	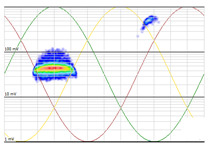	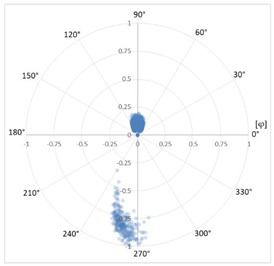	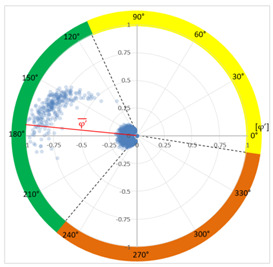
Corona Effect in phase 2	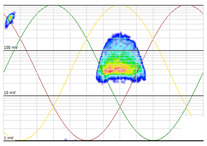	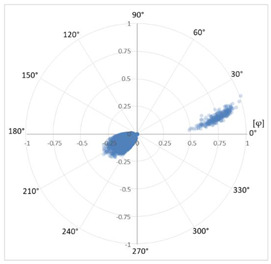	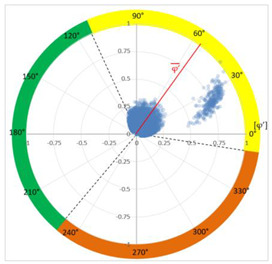
Corona Effect in phase 3	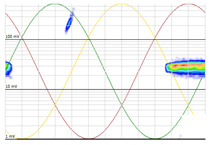	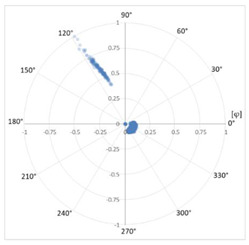	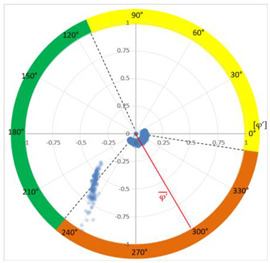

**Table 8:** Criterion polarity pattern according to ratio *R_positives_*.

PRPD Pattern	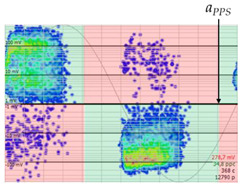	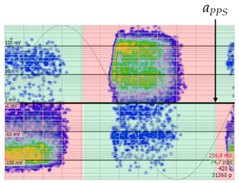	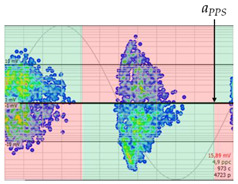
Ratio	*R_positives_* = 98%	*R_positives_* 3%	*R_positives_* = 58%
Polarity Criterion	*R_positives_* ≥ 60% POSITIVE	*R_positives_* ≤ 40% NEGATIVE	40% < *R_positives_* < 60% UNKNOWN

**Table 9:** Tests results.

Test	Clustering by Phase Selection	Polarity Location	PRPD of Pulses Related to Each Phase	AI Tool for PRPD Pattern Recognition
#1	Phase U 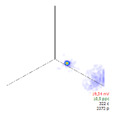	Negative: from GIS	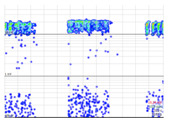	Floating Potential Defect type #7 (100%)
	Phase V 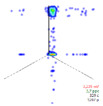	Positive: in the cable system	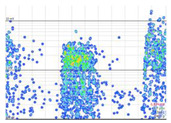	Void Defect type #16 (96%)
	Phase W 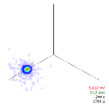	Positive: in the cable system	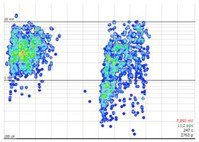	Surface between solid insulations (Defect type#15) (98%)
#2	Phase U 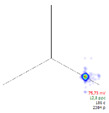	Negative: from GIS	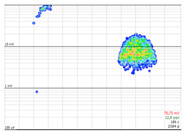	Corona in air (Defect type#1) (99%)
	Phase V 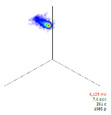	Positive: in the cable system	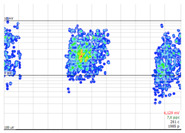	Surface between solid insulations (Defect type#15) (97%)
	Phase W 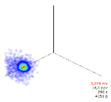	Positive: in the cable system	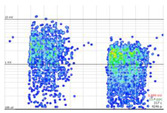	Void (Defect type #16) (98%)

**Table 10:** Clustering of the PD detected by the sensor on phase C at the MV cabinet P2.

Raw Measurement [RAW_P2_C]	Clustering Analyzing the Arrival Time of PD Pulses to the HFCT Sensors (Mean Value, *μ*, and Standard Deviation, *σ*). + Clustering Analyzing the Amplitude Ratio between the Three Phases Selection Orange Square [LOC_P2_C]			Additional Clustering of Using Wave form Parameters Selection Orange Box [VOID_P2_C]
No clustering tool is applied	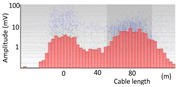 (*µ* = 78 m, *σ* = 11 m)	+	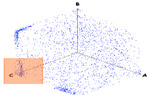 (0.1, 0.2, 1.0)	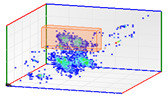 (31 s10^5^, 103 s10^5^, 3.5 MHz)
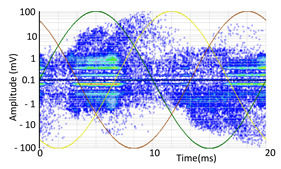	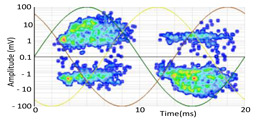 PRPD pattern of the PD pulses selected in the orange square			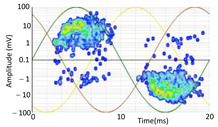 PRPD pattern of the PD pulses selected in the orange box
Total set of PD pulses detected with the HFCT sensor of phase C located on the MV cabinet P2.	PD cluster detected by the HFCT sensor (phase C) at MV cabinet P2 and with amplitude ratio between the three HFCT sensors concentrated in axis of sensor placed at phase C.			New cluster of internal void. Probability of 99%.

**Table 11:** Clustering PD detected by sensor on phase C at the MV cabinet P3.

Raw Measurement [RAW_P3_C]	Clustering Analyzing the Arrival Time of PD Pulses to the HFCT Sensors Located in P3 and P4 + Clustering Analyzing the Amplitude Ratio between the Three Phases P3 [LOC_P3_C]		
No clustering tool is applied	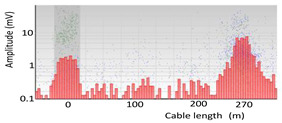 (*µ* = −1 m, *σ* = 12 m)	+	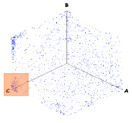 (0.05, 0.1, 1.0)
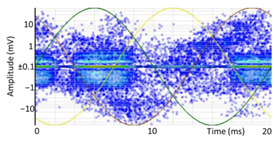	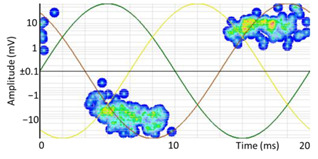 PRPD pattern of the PD pulses selected in the orange square of the amplitude ratio between the three phases.		
Total set of PD pulses detected with the HFCT sensor in phase C at the MV cabinet P3.	PD cluster of PD located at 0 m from the MV cabinet P3 and with amplitude ratio between the three HFCT sensors concentrated in axis of sensor at phase C. Void insulation defect with a probability of 98%.		

## Data Availability

Data are contained within the article.

## References

[1] (2010). Guidelines for Unconventional Partial Discharge Measurement.

[2] Govindarajan S., Morales A., Ardila-Rey J.A., Purushothaman N. (2023). A review on partial discharge diagnosis in cables: Theory, techniques, and trends. Measurement.

[3] Montanari G.C., Hebner R., Seri P., Ghosh R. (2020). Self-assessment of health conditions of electrical assets and grid components: A contribution to smart grids. IEEE Trans. Smart Grid.

[4] (2016). High Voltage Test Techniques—Measurement of Partial Discharges by Electromagnetic and Acoustic Methods.

[5] Vera C., Garnacho F., Klüss J., Mier C., Álvarez F., Lahti K., Khamlichi A., Elg A.P., Mor A.R., Arcones E. (2023). Validation of a Qualification Procedure Applied to the Verification of Partial Discharge Analysers Used for HVDC or HVAC Networks. Appl. Sci..

[6] Hu X., Siew W.H., Judd M.D., Peng X. (2017). Transfer function characterization for HFCTs used in partial discharge detection. IEEE Trans. Dielectr. Electr. Insul..

[7] Garnacho F., Álvarez F., Elg A.P., Mier C., Lahti K., Khamlichi A., Arcones E., Klüss J., Mor A., Vidal J.R. (2023). Metrological Qualification of PD Analysers for Insulation Diagnosis of HVDC and HVAC Grids. Sensors.

[8] Hussain G.A., Mahmood F. (2023). Review on Partial Discharge Diagnostic Techniques for High Voltage Equipment in Power Systems. IEEE Access.

[9] Mor A.R., Heredia L.C.C., Harmsen D.A., Muñoz F.A. (2018). A new design of a test platform for testing multiple partial discharge sources. Int. J. Electr. Power Energy Syst..

[10] 19ENG02 Future-Energy from the EMPIR Program Co-Financed by the Participating States and from the European Union’s Horizon 2020 Research and Innovation Program. https://www.euramet.org/research-innovation/search-research-projects/details?tx_eurametctcp_project%5Bproject%5D=1698&cHash=4dcb5896e17fe25f799b0c8ae8d13130.

[11] Mas’ud A.A., Albarracín R., Ardila-Rey J.A., Muhammad-Sukki F., Illias H.A., Bani N.A., Munir A.B. (2016). Artificial Neural Network Application for Partial Discharge Recognition: Survey and Future Directions. Energies.

[12] Stone G.C., Cavallini A., Behrmann G., Serafino C.A. (2023). Practical Partial Discharge Measurement on Electrical Equipment.

[13] Morshuis P., Montanari G.C., Fornasari L. Partial discharge diagnostics—Critical steps towards on-line monitoring. Proceedings of the 2014 IEEE PES T&D Conference and Exposition.

[14] Tozzi M., Salsi A., Busi M., Montanari G.C., Cavallini A., Hart P.M. Permanent PD monitoring for generators: Smart alarm management. Proceedings of the 2011 IEEE PES Innovative Smart Grid Technologies.

[15] Khamlichi A., Garnacho F., Simón P. (2023). New Synthetic Partial Discharge Calibrator for Qualification of Partial Discharge Analysers for Insulation Diagnosis of HVDC and HVAC Grids. Sensors.

[16] Arcones E., Álvarez F., Khamlichi A., Garnacho F. (2024). Scale Modular Test Platform for the Characterization of PD Measuring Systems Using HFCT Sensors. Sensors.

[17] Shafiq M., Hussain G.A., Kütt L., Elkalashy N.I., Lehtonen M. (2015). Partial discharge diagnostic system for smart distribution networks using directionally calibrated induction sensors. Electr. Power Syst. Res..

[18] (2006). High-Voltage Test Techniques-Partial Discharge Measurements.

[19] Koltunowicz W., Plath R. (2008). Synchronous multi-channel PD measurements. IEEE Trans. Dielectr. Electr. Insul..

[20] Lalitha E.M., Satish L. (2000). Wavelet analysis for classification of multi-source PD patterns. IEEE Trans. Dielectr. Electr. Insul..

[21] Zhou X., Zhou C., Kemp I.J. (2005). An improved methodology for application of wavelet transform to partial discharge measurement denoising. IEEE Trans. Dielectr. Electr. Insul..

[22] Qiao M., Wang H., Jiang J., Li C., Gong W., Li Q. Partial Discharge Denoising Method Based on Improved Wavelet Threshold Optimized by Double Chain Quantum Genetic Algorithm. Proceedings of the 2020 IEEE International Conference on High Voltage Engineering and Application (ICHVE).

[23] Raymond W.J.K., Xin C.W., Kin L.W., Illias H.A. (2021). Noise invariant partial discharge classification based on convolutional neural network. Measurement.

[24] Andrade G.O., Avelar A.S.O., Mota H.O. Comparative Analysis of Partial Discharge Denoising Techniques. Proceedings of the Electrical Insulation Conference and Electrical Manufacturing & Coil Winding Conference.

[25] Song X., Zhou C., Hepburn D.M. An Algorithm for Indentifying the Arrival Time of PD Pulses for PD Source Location. Proceedings of the 2008 Annual Report Conference on Electrical Insulation and Dielectric Phenomena.

[26] Wild M., Tenbohlen S., Gulski E., Jongen R., de Vries F. Practical aspects of PD localization for long length power cables. Proceedings of the IEEE Electrical Insulation Conference (EIC).

[27] Bakar A.A., Yii C.C., Fern C.K., Pin Y.H., Lago H., Rohani M.N.K.H. (2023). A Comparison of Double-End Partial Discharge Localization Algorithms in Power Cables. Energies.

[28] Robles G., Shafiq M., Martínez-Tarifa J.M. (2019). Multiple partial discharge source localization in power cables through power spectral separation and time-domain reflectometry. IEEE Trans. Instrum. Meas..

[29] Álvarez F., Garnacho F., Ortego J., Sánchez-Urán M. (2015). Application of HFCT and UHF sensors in on-line partial dis-charge measurements for insulation diagnosis of high voltage equipment. Sensors.

[30] Sánchez A., Garnacho F. (2023). Requirements of Artificial Intelligence Platform addressed to Automatic Assessment of Insulation Condition of Indoor and Outdoor Installations through Partial Discharge Monitoring. CIGRE Sci. Eng. A Scopus Regist. Mag. (CSE).

[31] Castro L., Mor A.R. (2019). Density-based clustering methods for unsupervised separation of partial discharge sources. Int. J. Electr. Power. Energy Syst..

[32] Firuzi K., Vakilian M., Darabad V.P., Phung B.T., Blackburn T.R. (2017). A novel method for differentiating and clustering multiple partial discharge sources using S transform and bag of words feature. IEEE Trans. Dielectr. Electr. Insul..

[33] Alvarez F., Ortego J., Garnacho F., Sanchez-Uran M.A. (2016). A Clustering Technique for Partial Discharge and Noise Sources Identification in Power Cables by Means of Waveform Parameters. IEEE Trans. Dielectr. Electr. Insul..

[34] Mor A.R., Heredia L.C.C., Muñoz F.A. (2017). New clustering techniques based on current peak value, charge and energy calculations for separation of partial discharge sources. IEEE Trans. Dielectr. Electr. Insul..

[35] Cong Z., Gang W., Dong G., Wei Y., Kai W., Ming L.U., Ying L., Yajin L.I. (2021). Partial discharge pattern recognition based on convolutional neural network. Adv. Technol. Electr. Eng. Energy.

[36] Mazroua A.A., Salama M.M.A., Bartnikas R. (1993). PD pattern recognition with neural networks using the multilayer perceptron technique. IEEE Trans. Electr. Insul..

[37] Ding B., Liu J., Chen J., Zhou Y., Zhou Z., Zhang Y. Pattern recognition of partial discharge based on deep learning. Proceedings of the 16th IET International Conference on AC and DC Power Transmission.

[38] Firuzi K., Vakilian M., Phung B.T., Trevor R. (2018). Partial Discharges Pattern Recognition of Transformer Defect Model by LBP & HOG Features. IEEE Trans. Power Deliv..

[39] Araújo R.C.F., Oliveira R.M.S., Barros F.J.B. (2022). Automatic PRPD Image Recognition of Multiple Simultaneous Partial Discharge Sources in On-Line Hydro-Generator Stator Bars. Energies.

[40] Abubakar A., Zachariad C. (2024). Phase-Resolved Partial Discharge (PRPD) Pattern Recognition Using Image Processing Template Matching. Sensors.

[41] Florkowski M. (2021). Anomaly Detection, Trend Evolution, and Feature Extraction in Partial Discharge Patterns. Energies.

[42] Garnacho F., Khamalichi A. (2022). Insulated, Shielded Partial Discharge Sensor Device of the High-Frequency Current Transformer (HFCT) Type, with High Gain and Low Distortion, and with Self-Check System for High-Voltage Equipment and. Installations. Patent.

